# Association between daytime napping duration and depression in middle-aged and elderly Chinese: evidence from the China Health and Retirement Longitudinal Study (CHARLS): A cross-sectional study in China

**DOI:** 10.1097/MD.0000000000022686

**Published:** 2020-10-23

**Authors:** Baoming Xie, Jinhuan Wang, Xiaoyu Li, Jingyuan Zhang, Miaomiao Chen

**Affiliations:** a The Affiliated Yantai Yuhuangding Hospital of Qingdao University, Yantai, Shandong; b Laboratory of Innovation, Basic Medical Experimental Teaching Centre, Chongqing Medical University, Chongqing, China.

**Keywords:** daytime napping, depression, middle-aged and elderly

## Abstract

The effect of the afternoon napping duration on the risk of depression has not been well established, particularly with regard to sex and age differences. The present study examines the association between afternoon napping duration and depression stratified by sex and age among Chinese adults aged 45 years or older.

The 2011 to 2012 survey of the China Health and Retirement Longitudinal Study was utilized, including 5746 participants. We conducted logistic regression with the overall sample and subjects stratified by sex and age.

Elderly men with short napping (<30 minutes) had lower odds of having depression symptoms compared with those with no napping group (OR = 0.66, 95% CI = 0.44–0.97). In addition, the finding indicated that middle-aged women with long napping (≥90 min) had a marginally significant difference than those in reference, which showed a negative effect on depression (OR = 0.72, 95% CI = 0.51–1.01).

Our findings revealed that extended daytime napping duration can decrease the risk of depression status among middle and elderly people. Moreover, relevant promotion measures should be adopted, such as a suitable rest environment and regular napping habits. The potential mechanism should be clarified by a longitudinal survey to examine the specific causality.

## 1 Introduction

Depression in aging adults is a serious public concern worldwide because it was detected to be positively associated with cardiac morbidity and mortality, suicide, and loss of physical and/or social function.^[1]^ Previous studies have documented that depression may be linked to chronic noncommunicable diseases, such as hypertension, diabetes, and stroke.^[2–4]^ In addition, Birney et al^[5]^ posited that depression would rise to the second leading cause of disability by 2020. The World Health Organization estimated that more than 121 million older adults in 2005 and 350 million people of all ages in 2013 suffered from depression globally.^[6,7]^ In China, the prevalence of depression has dramatically increased over the years.^[8]^ According to the data of the Sixth Census in 2010, the population aged 60 and overreached 178 million, accounting for 13.26% of the total population.^[9]^ By the end of 2016, the population aged 60 and over had reached 16.7%.^[10]^ The number of elderly aged 60 and above in China is increasing annually, making China the largest elderly population in the world. Therefore, the prevention and treatment of depression have drawn great attention in the region of public health and management.

Sleep and mental health have been associated with depression in epidemiological and clinical studies.^[11,12]^ The growing epidemiological literature indicates that short nighttime sleep duration was an independent risk factor of depression symptoms incidence and persistence.^[13,14]^ Although the association between depression and sleep duration has been previously evaluated in several studies, evidence on the afternoon napping is limited.

Daytime napping is traditionally regarded as a part of a health-promoting lifestyle in China.^[15]^ However, the effect of daytime napping has inconsistent results for decades. Previous studies have explored the positive effects of daytime napping on improving individual cognition and memory function and promoting alertness and well-being.^[16,17]^ In addition, some studies have found that extended nap is associated with increased risks of diabetes, hypertension, and all-cause mortality among older and youth populations.^[18–20]^ With regard to the relationship between daytime napping and depression, a previous study in Australia revealed that long and frequent daytime naps are linked to high levels of depression symptoms among older people with dementia.^[21]^ Another cohort study with the Chinese reported that a significantly positive association is observed between depression and afternoon napping among 0.5 million adults. However, no analyses were performed on age- and sex-specific effects.^[22]^

Based on the data from China Health and Retirement Longitudinal Study (CHARLS), the present study aims to explore the potential association between daytime napping and depression symptoms among middle-aged and elderly adults and detect the sex- and age-specific difference among subjects.

## 2 Materials and methods

### 2.1 Study population

Peking University established the CHARLS, a nationwide prospective cohort based on multistage probability sampling, to evaluate the physical and psychological health of the population aged 45 and above. This project, uncorking from 2011, has recruited 17,708 participants so far. Detailed information related to CHARLS has been reported elsewhere.^[23]^ Participants aged 45 years or older in the baseline survey in 2011 to 2012 were enrolled in the present study, whereas those with missing data on depression symptoms or daytime napping were excluded. The ethics of CHARLS has been approved by the Ethical Review Committee of Peking University, and written informed consent was provided by all participants.

### 2.2 Data collection

The trained volunteers adopted a semistructured questionnaire to collect data including individual demographic characteristics, health status, lifestyle, and information of the blood sample from the follow-up surveys through a face-to-face interview every 2 years. Height, weight, and waist circumference were measured using the standardized instruments. Body mass index (BMI) was calculated as weight in kilograms divided by height in meters squared. Blood pressure was measured 3 times, and the mean value of the 3 measurements was employed for analysis. Blood samples were harvested by trained medical staff and stored at the Chinese Center for Disease Control and Prevention. Diabetes was defined as a fasting glucose level of ≥126 mm/dL (7.0 mmol/L), self-reported use of diabetes medication, or self-reported diagnosis of diabetes.^[24]^ Dyslipidemia was identified using decreased High-density lipoprotein cholesterol concentration (men <40 mg/dL, women <50 mg/dL), elevated LDL-C concentration of >130 mg/dL, triglyceride concentration of ≥150 mg/dL, or a self-reported diagnosis of dyslipidemia.^[25]^ Those with a C-reactive protein (CRP) concentration of ≥3 mg/dL were considered high CRP.^[26]^

### 2.3 Assessment of depression

The CHARLS measured the symptoms of depression using the 10-term CESD. The CESD questionnaire included the following: I was bothered by things that do not usually bother me. I had trouble keeping my mind on what I was doing. I felt depressed. I felt everything I did was an effort. I felt hopeful about the future. I felt fearful. My sleep was restless. I was happy. I felt lonely. I could not get “going.” Each item was scored on a 4-point scale: “rarely or none of the time (<1 day),” “some or a little of the time (1–2 days)” to “occasionally or a moderate amount of the time (3–4 days),” or “most or all of the time (5–7 days).” The Cronbach's ɑ of CESD was 0.84 with the CHARLS sample. The cut-off point of depression status score was 10.^[27]^

### 2.4 Assessment of daytime napping duration

Sleep duration at night was evaluated by asking, “During the past month, how many hours of actual sleep did you get at night (average hours per night)? (This may be shorter than the number of hours you spend in bed.)” We categorized night sleep duration into 3 groups: <7, 7 to 8, and ≥9 hours.^[28]^ Afternoon napping duration was assessed by asking, “During the past month, how long did you take a nap after lunch in general?” Regular daytime napping was classified into 5 groups: 0, <30, 30 to 59, 60 to 89, and ≥90 minutes.^[29]^

### 2.5 Statistical analysis

The basic characteristics of the participants included demographic, clinical, and behavioral variables. Differences in these characteristics with depression status (normal vs depression) were examined using the Wilcoxon rank sum test for continuous variables and the *χ*^2^ test for categorical variables. Differences in age, sex, and nighttime sleep duration by daytime napping duration were examined using the Kruskal–Wallis rank test or *χ*^2^ test. Two logistic regression models were used to further examine the sex- and age-specific association between the daytime napping duration and depression. Model 1 was adjusted for demographic characteristics (age groups, sex, education, marital status, region), and Model 2 for demographic characteristics and clinical and behavioral variables (BMI, waist circumference, smoking status, alcohol drinking, nighttime sleep duration, diabetes status, dyslipidemia, high CRP, and depression). All statistical tests were 2-sided and a *P* value less than .05 was considered statistically significant. Data were managed and analyzed using Stata 15.0 (Stata Corp LP, College Station, TX).

## 3 Results

A total of 5746 participants were employed in our study. As shown in Table 1, our sample had 52.51% of participants aged 45 to 59 years and 47.49% of subjects over 60 years of age. Half of the people were female (51.34%), and the majority lived in rural areas (82.21%) and were married (81.45%) with primary education (43.26%). Approximately 42.16% of the sample suffered depression symptoms. As for daytime napping duration, 46.12% of participants were reported never napping, 9.80% was below 30 minutes, 7.83% was between 30 and 59 minutes, 22.12% ranged from 60 to 89 minutes, and 14.13% was over 90 minutes. Compared with middle-aged adults, older adults had a high proportion of depression symptoms (49.15% vs 50.85%). Furthermore, the proportions of depression symptoms in females (59.72%) were higher than males (40.28%). Sample characteristics were compared between normal and depression groups. Interestingly, compared with the former, the latter had less consumption of tobacco and alcohol but with more proportion of overweight and obese.

**Table 1:**
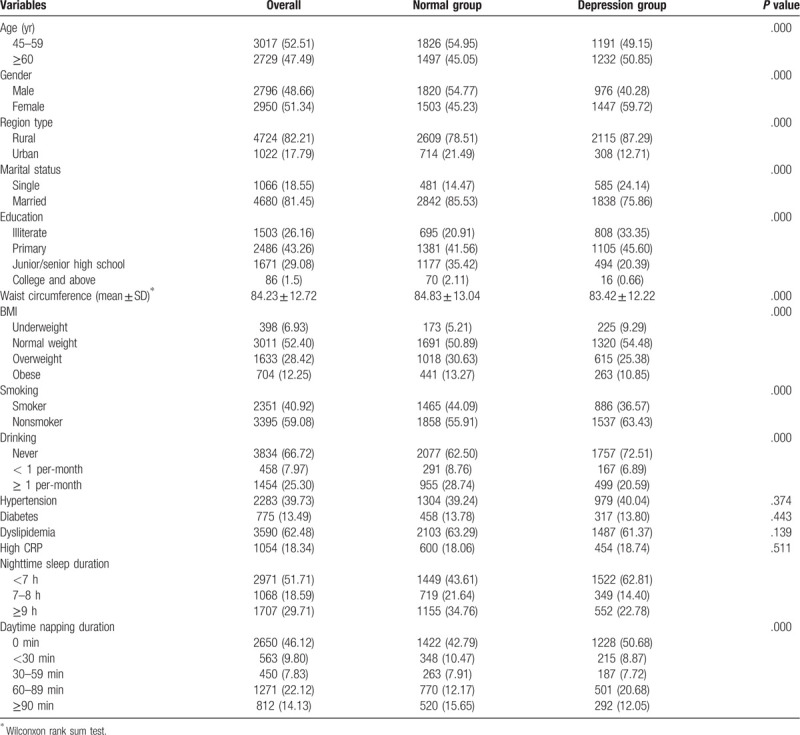
The characteristics of study variables.

We examined alterations in gender (*P* < .001), ages (*P* < .05), and nighttime sleep duration (*P* < .001) across daytime napping time in Table 2. A high proportion who reported no napping was middle ages (54.49%), and those reported extremely high duration was the majority of older (51.85%). According to reports, the proportion of females without a nap was high (57.70%), and the proportion of males with ≥90 minutes napping was also high (59.11%). People in the ≥90 minutes group showed a longer nighttime sleep duration than other types of napping groups (≥90 minutes napping group: 6.70 ± 1.92).

**Table 2:**
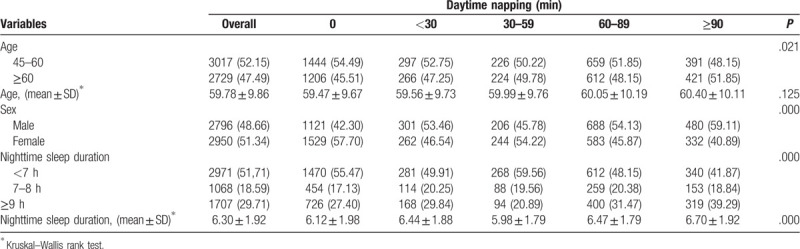
Age, sex, and nighttime sleep duration according to afternoon napping group.

As shown in Table 3, we tested the association between daytime napping duration and depression symptoms in the final analysis. After controlling the confounding variables, people in ≥90 minutes napping group had decreased odds of having depression symptoms compared with those in the never napping group (OR = 0.90, 95% CI = 0.68–0.96). In addition, females had higher odds of having depression symptoms than those in the male group (OR = 1.68, 95% CI = 1.42–1.98). No significant differences in having depression symptoms were observed between males and females.

**Table 3:**
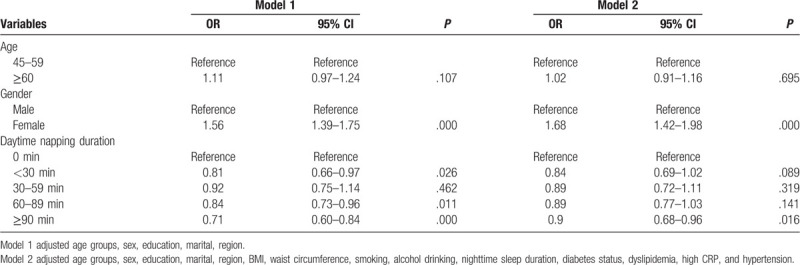
Associations between daytime napping duration and depression.

A fully adjusted model was conducted revealing that female in above 90 minutes napping group was less likely to suffer depression symptoms (OR = 0.76, 95% CI = 0.59–0.98) to analyze the variation of sex and age. Moreover, male in short daytime napping (<30 minutes) was a protective factor for depression symptoms (OR = 0.70, 95% CI = 0.53–0.94). However, no significant difference was reported when compared with the younger and elderly group in other types of daytime napping duration. In addition, we categorized 4 age- and gender-specific groups, as follows: middle-aged men group, older adult men group, middle-aged women group, and older women group. As shown in Table 4, older men in below 30 minutes group had lower odds of having depression symptoms compared with those in no napping group (OR = 0.66, 95% CI = 0.44–0.97). In addition, the results indicated that middle-aged women in more than 90 minutes group had a marginally significant difference than those in reference and showed a negative effect (OR = 0.72, 95% CI = 0.51–1.01).

**Table 4:**
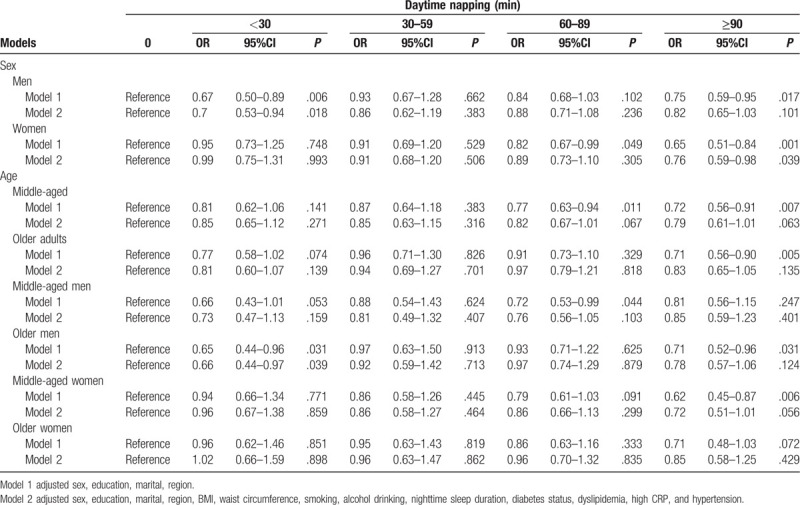
Associations between afternoon napping duration and depression by sex and age.

## 4 Discussion

The age- and gender-specific difference in the association between daytime napping duration and depression was examined by analyzing the national baseline datasets from CHARLS among adults over 45 years. This current study found a negative association between depression symptoms and daytime napping in the overall sample, and the strength of the association varied with age and gender. In the men group, short daytime napping (<30 minutes) was negatively associated with depression symptoms, specifically in older men. In the women group, long daytime napping (>90 minutes) was conversely connected with the risk of depression symptoms, particularly in middle-aged women showing a marginal significance. Our finding further unveiled the sex- and age-specific relationship between daytime napping and depression symptoms in the Chinese aging population.

Previous study has reported the J-curve relationship between afternoon napping duration and incidence of type 2 diabetes and cardiovascular disease, and the linear relationship between afternoon napping duration and all-cause mortality.^[30]^ However, studies specializing in daytime napping duration and depression are limited. As for this relationship, researchers may focus on the effect of daytime napping duration on patients with abnormal mental state. One longitudinal study indicated that depressed participants with longer midday napping were tied to a low risk of persistent depression symptoms.^[13]^ Australian research disclosed that long and frequent daytime naps were associated with high levels of depression symptoms among older people.^[21]^ The power of a single study was limited possibly because of the small sample size and leading to paradoxical results. For example, in our study, never napping accounted for 46.12%, but short and moderate napping were only 9.80% and 7.83%, respectively.

Historical studies conducted in China present some discrepancies. Liu et al^[22]^ reported that having daytime napping sustains high odds of depression for females (OR = 1.15, 95% CI: 1.01–1.31) and males (OR = 1.42, 95% CI: 1.18–1.71). This conclusion is consistent with Cross's findings^[21]^ but not ours. The latent reasons may connect with the way to define subgroups. In Liu's study, daytime napping is just parted into Yes or No, which limits understanding of the full spectrum. Our study also indicates that the length of daytime napping matters. Not all subgroups in the present study display protective effects. Thus, further reanalysis of Liu’ data may reveal some new findings. Some studies may support our findings. The result from Li et al ^[31]^ reveals that 30 to 90 minutes afternoon napping enjoys better cognitive functions than non-nappers and short nappers (<30 minutes), which could provide better mental quality for the aged.

The mechanisms of the association between daytime napping and depression have not been elucidated. Several reported mechanisms may clarify that increased afternoon daytime napping duration reduces the risk of the prevalence of depression symptoms. First, some evidence has stated that short nighttime sleep duration may increase great odds of depression.^[32]^ High odds of depression in insomnia patients attract much of the researcher's attention,^[33]^ reflecting the vital importance of sleep quality. Effective daytime napping habits may provide them another sleep opportunity, thereby enhancing their sleep quality and further improving mental quality. Second, for many elderly people with physical tiredness and psychological fatigue,^[34]^ never daytime napping may increase the risk of circadian disorder and the alteration of hormone levels.

This study comprises several strengths. First, studies examining the sex- and age-related associations between daytime napping duration and depression are limited. Second, few studies look into the interactions of daytime napping duration and risk factors including sex, age, BMI, and personal history of diabetes and hypertension on the prevalence of depression. Third, the CHARLS was conducted using a complex, multistage, probability sampling design, so our findings may be seen as convincing. However, several limitations should be considered. First, the cross-sectional design curtails the ability in addressing causal relationships between daytime napping duration and depression. Second, participants included in the present study were middle-aged and elderly Chinese participants, so the findings might not be generalized to other populations. Third, we measured daytime napping duration only through self-reported, which may lead to minor inaccuracies. Fourth, the assessment of depression was concluded with the 10-item CES-D, which may lead to minor inaccuracies. Fifth, previous studies have examined the independent effect of excessive sleepiness on obesity.^[35]^ Hence, further cohort study should be conducted to explore the causality mechanism among napping, depression, and abnormal weight.

## 5 Conclusions

In a large nationally representative sample of middle-aged and older Chinese, long afternoon napping (≥90 minutes) was associated with depression in middle-aged women, and a short afternoon napping (<30 minutes) was associated with depression in older men. However, future longitudinal studies are needed to confirm the association. Interdisciplinary collaboration among researchers, cardiovascular, and sleep specialists is needed to further explore the relationship between afternoon napping and depression and the possible mechanisms behind the association.

## Acknowledgments

The authors voice their thanks to CHARLS office.

## Author contributions

Conceptualization, BX, JHW, and MMC; Methodology, XYL and JYZ; Validation, XYL; Formal Analysis, BX and JHW; Data Curation, XYL; Writing-Original Draft Preparation, BX and JHW; Writing-Review and Editing, MMC; Visualization, BX and JHW.

**Data curation:** Xiaoyu Li, Jingyuan Zhang.

**Formal analysis:** Xiaoyu Li.

**Methodology:** Xiaoyu Li.

**Project administration:** Jinhuan Wang.

**Resources:** Jingyuan Zhang.

**Software:** Jinhuan Wang, Jingyuan Zhang.

**Validation:** miaomiao chen.

**Visualization:** miaomiao chen.

**Writing – original draft:** Baoming Xie.

**Writing – review & editing:** Baoming Xie, Jinhuan Wang, miaomiao chen.
